# Ultra-Processed Food as Mediator of the Association between Birthweight and Childhood Body Weight Outcomes: A Retrospective Cohort Study

**DOI:** 10.3390/nu15194178

**Published:** 2023-09-27

**Authors:** Min Hou, Chao Qiu

**Affiliations:** 1 School of Public Health, College of Medicine, Shanghai Jiao Tong University, 227 Chongqing South Road, Shanghai 200025, China; 2 College of Humanities, Jiangnan University, 1800 Lihu Road, Wuxi 214122, China

**Keywords:** body composition, children, high birthweight, interaction, low birthweight, ultra-processed food

## Abstract

Previous studies have shown conflicting findings regarding the association between birthweight and childhood adiposity. We aimed to explore the interaction between ultra-processed food (UPF) and birthweight and its associations with bodyweight markers. The retrospective analysis of data from a Multicity Cohort Study across eastern China was conducted. UPF was computed as percentage of the energy intake and categorized into quartiles. Birthweight was categorized into low (LBW), normal (NBW) and high (HBW). The BMI z-score was calculated using the lambda-mu-sigma method. The sex- and age-specific BMI cutoff points were used to define weight status. Generalized linear models were used to examine modification effects and were performed after adjustment for covariates. The mean percentage of energy intake from UPF was 27.7% among 1370 children. Of all children, 2.3% and 21.4% were born with LBW and HBW, respectively. HBW was a permanent risk for high BMI measures, while LBW was associated with increased BMI measurements only by the addition of the interaction term. The subgroup analysis revealed that HBW and LBW were positively associated with BMI measurements in the lowest UPF intake (Q1), while HBW was related to high BMI measures in Q4. Our findings support efforts to recommend limiting UPF intake, especially for LBW children.

## 1. Introduction

Childhood obesity, or overweight, is an important risk factor for health problems such as cardiovascular diseases, chronic conditions, psychosocial complications and cancers [1] and likely continues into adulthood [2]. Its elevated prevalence in both developed and developing countries places a burden on healthcare expenditure and is a major threat to public health throughout the world [1,3]. With the economic growth and urbanization in China during the past four decades, the overweight and obesity rate has been rising four-fold in children aged 6–17 years, with the largest number in the world [4]. The failure of currently available treatments for overweight and obesity has resulted in an emphasis on effective approaches to improve adiposity, which has been considered a public health challenge [5]. A better understanding of the factors related to childhood-weight-related problems is therefore of paramount importance.

A number of studies provide evidence to support the idea that diet is an important factor in regulating growth, metabolism and obesity risk [6,7]. Importantly, with rapid economic development, changes in the food system, such as increased intakes of processed foods, are hypothesized to play a key role in the epidemic of pediatric overweight and obesity worldwide [4]. The NOVA classification is a prominent approach to food classification that identifies dietary patterns based on the extent of food processing [8]. Ultra-processed food (UPF) refers to ready-to-eat or ready-to-heat foods that are manufactured with little, if any, whole foods and substances refined or extracted from foods and often include food additives, among which are mostly of exclusive industrial use [9]. UPF is in general energy-dense, high in fat, added sugar and salt, but low in fiber content, being considered as a poorer nutritional quality to convey adverse health effects, such as obesity [10,11].

UPF products are visually appealing and aggressively advertised [9] and have gradually displaced traditional dietary patterns based on fresh and minimally processed foods [12]. The National Health and Nutrition Examination Surveys presented a significant increase from 61.4% to 67.0% of daily calorie intake in UPF consumption among US youths from 1999 to 2018 [13]. Children 4–10 years old consumed 65.4% of their daily energy intake from UPF in the UK [14]. In a cross-sectional study of 428 children aged 6 to 36 months from Shijiazhuang, China, the percentage of UPF consumption relative to total food intake was between 73.8% and 98.2% [15]. It was also reported that UPF contributed to 41.8% of the total energy intake in adults aged 18–90 years in Tianjin, China [16]. These findings indicate that UPF consumption is variable across age and ethnic groups of children. Most importantly, there is a lack of available data on UPF consumption among Chinese school-aged children.

The emerging evidence supports that birth weight is associated with later BMI [17,18], BMI z-score [19,20] and risk of overweight or obesity among children [21,22,23]. The epidemiological studies have consistently identified that high birth weight (HBW) is related to increased BMI [17] and the risk of overweight or obesity in childhood [24,25,26,27]. However, there were conflicting findings across different study groups, for the associations between low birth weight (LBW) and the risk of childhood obesity [21,28,29,30,31,32]. For instance, some studies reported negative relationships of LBW with the risk of overweight or obesity [28,29,31,32]. However, a large cross-sectional study including 70,284 children aged 3–12 years in China reported that LBW was associated with severe obesity rather than overweight or obesity [33]. Data collected from 12 countries revealed a positive association between LBW and BMI for age z-score only in boys aged 9–11 years [19]. In other studies, no association was found between LBW and the risk of overweight and obesity or BMI measurements [18,21]. Thus, a possible missing link in the observed relation between birth weight, especially LBW and childhood body composition trajectories, remains.

Recent systematic reviews and meta-analyses provide consistent evidence suggesting a positive association between UPF and overweight and obesity in children [34,35]. However, there is a paucity of research examining the impacts of UPF intake on body composition among children with different birth weights. It is also important to understand the impacts of UPF consumption on the relationship between birth weight and childhood body composition. We hypothesized that birth weight is associated with greater childhood BMI measurements and the risk of overweight and obesity, and the relationships vary by childhood UPF consumption. We aimed to test this hypothesis by using our population-based cohort with data on prenatal measures and dietary intake among schoolchildren between 7 and 16 y of age in Eastern China.

## 2. Materials and Methods

### 2.1. Study Design and Participants

**Figure 1:**
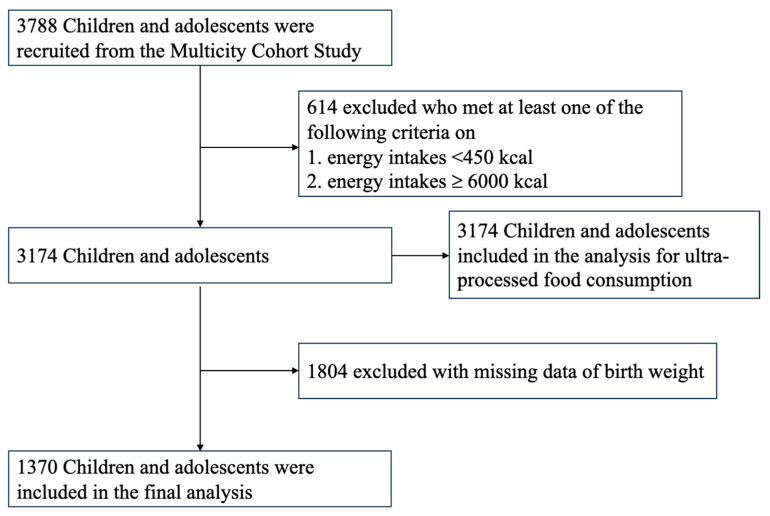
Flowchart of the study cohort for analysis.

A retrospective cohort study was conducted on 3788 available children and adolescents from Shanghai, Jiaxing, Dezhou, Suqian, Taizhou and Fuyang across eastern China between 2019 and 2021. We selected cities with upper, moderate and lower levels of socioeconomic status based on data reported in 2018 by the National Bureau of Statistics of China [32] to provide a regional representative database. Children were randomly selected from each grade in nine primary and secondary private and public schools based on using random numbers via computer systems. Children who had serious organ disease, abnormal physical development or physical impairments were excluded from this study. Parents and children were informed of the study by class teachers, and those children whose parents provided written informed consent were recruited. Children aged 12 years or older also provided the simple version of written informed consent. The structured questionnaire was required to be completed to collect information on demographic characteristics, lifestyle behavior, dietary intake, obstetrics and medical history. A subgroup analysis of the dataset from this observational study was performed by selecting participants who had birth record data (*n* = 1370) (Figure 1). Data were analyzed from 1 January 2022 to 31 May 2022. The study was approved by the Ethics Committee of the School of Public Health of Shanghai Jiao Tong University for Human Subject Research, and all parents gave written informed consent (No. SJUPN-201815).

### 2.2. Socioeconomic and Anthropometric Measures

The structured questionnaire included information on the child’s sex, age, body weight in kilograms, height in centimeters (without shoes), birth weight in grams, delivery mode, breastfeeding history, physical activity, delivery mode, as well as the maternal weight, height, education, gestational age at birth and family income. The maternal and child’s demographic characteristics and lifestyle behaviors were reported by mothers. Clinical information, including birth weight, was obtained based on birth records. The physical activity section of the questionnaire included information on moderate physical activity (e.g., walking) and vigorous physical activity (e.g., running, basketball/soccer, swimming, cycling, etc.). Duration of physical activity per day was categorized as ‘less than 30 min’, ‘30–60 min’ and ‘more than 60 min’. Maternal education was categorized as ‘Secondary high school and lower,’ ‘High school or equivalent,’ and ‘Bachelor degree and higher’. Annual household income was categorized as ‘less than 80,000 RMB’, ‘80,000–150,000 RMB’ and ‘more than 150,000 RMB’. Breastfeeding history was categorized as ‘never’, ‘less than 6 months’ and ‘more than 6 months’. Vaginal delivery included spontaneous, assisted breech, breech extraction, vacuum extraction and forceps delivery, while cesarean delivery was composed of elective and emergency cesarean delivery [36]. Gestational age at birth was categorized as preterm or early term (gestational age relative to less than 38 completed weeks), full term (gestational age relative to 39–40 completed weeks) and late or post-term (gestational age relative to more than 41 weeks).

Mothers were encouraged to report their weight and height following instructions of weight and height measurements at home. Many studies suggest that self-reported height and weight were in agreement with the measured data [37]. BMI was calculated as weight divided by height squared (m^2^). A child’s BMI age and sex z-score was defined as BMI-standard deviation score (SDS) with the LMS (lambda-mu-sigma) method [38], based on the BMI growth curves for Chinese children and adolescents [39]. The sex- and age-specific BMI cutoff points recommended by the Working Group for Obesity in China (WGOC) [40] were used to define underweight, normal-weight, overweight and obesity among children and adolescents, which were consistent with the Eastern Asian ethnic characteristics of body fat growth [41]. Maternal BMI was categorized as underweight (<18.5 kg/m^2^), normal weight (18.5–23.9 kg/m^2^), overweight (24.0–27.9 kg/m^2^) and obesity (≥28.0 kg/m^2^).

### 2.3. Ultra-Processed Food Groups

This study collected dietary data with a 142-item food frequency questionnaire (FFQ). Frequencies of food consumption were measured in nine categories, ranging from never to >3 times/d. Children and a parent who was familiar with the child’s diet habits responded to the FFQ. According to NOVA food classification, the foods were classified and categorized into four food groups based on the extent and purpose of industrial food processing (Supplemental Table S1) [8]. Among these groups, ultra-processed food (UPF) is a type of food and drink product that is made from industrial substances is manufactured through a series of complex industrial process and often contains cosmetic food additives [8]. In this study, they were industrially processed grain with added preservatives or emulsifiers, fast foods, sweet snacks and sweets, industrial meat snacks, savory snacks, sugar-sweetened beverages, dairy foods and products, fast-food potato products and soy products. The percentage of UPF consumption was computed as the proportion of UPF consumed in the total daily food intake. The percentage of energy intake from UPF was computed as the ratio of the mean energy from the UPF group over the mean total energy intake, expressed as a percentage. Further calculations were made to obtain the percentage of subgroups of UPF consumption and the percentage of energy intake from subgroups of UPF. Individuals’ UPF consumption, expressed as a percentage of daily UPF consumption, was categorized into quartiles based on the cutoff points derived from dietary data of UPF consumption, expressed as a percentage. Individuals’ percentage of daily energy intake from UPF was categorized into quartiles based on the cutoff points derived from the dietary data of daily energy intake from UPF, expressed as a percentage.

The FFQ has been validated in a subsample of 120 children from the study population conducted by trained interviewers among children. The spearman correlation coefficients between the FFQ and the mean of four 1-week diet records collected at approximately three-month intervals were 0.53 for total daily energy intake, 0.52 for protein intake, 0.49 for intake of total fats and 0.57 for carbohydrates intake.

### 2.4. Study Covariates

Covariates included children’s age at assessment, sex (male or female), physical activity (<30 min/d, 30 to 60 min/d or ≥60 min/d), total daily energy intake (continuous), delivery mode (vaginal delivery, or caesarean delivery), breastfeeding (Never, <6 months, or ≥6 months), maternal education (less than high school, high school and general equivalency, college graduate or postgraduate and above), maternal BMI (<18.5, 18.5 to 24 or 24 to 28, ≥28), gestational age at birth (preterm or early term, full term or late or post-term) and annual family income (<80,000 RMB, 80,000 to 150,000 RMB or ≥150,000 RMB).

### 2.5. Statistical Analysis

Means and standard deviations (SD) were used for continuous variables, and frequencies and percentages for categorical variables or ordinal variables, within strata defined by the quartiles of UPF consumption. The normal distributions for continuous variables were assessed using the Shapiro–Wilk test. UPF consumption, expressed as the percentage of daily consumption of UPF, and the percentage of daily energy intake from UPF was equally divided into quartiles. The first quartile (Q1) was set as the lowest daily UPF consumption group or the lowest energy intake from UPF group. The fourth quartile (Q4) was set as the highest daily UPF consumption group or the lowest energy intake from UPF group. To examine differences in characteristics between the quartiles of UPF consumption, χ^2^ tests and Kruskal–Wallis 1-way ANOVA tests were used where appropriate. Fisher’s exact test was used for categorical or ordinal variables with more than 20% of the expected cell frequencies being less than 5%.

Generalized linear models with an identity function were conducted to determine associations between birth weight, UPF consumption and BMI measures, using normal birth weight (NBW) or the lowest intake of UPF consumption (Q1) as the reference category. In addition, generalized linear models and logistic regression were used to assess risk ratios of birth weight and UPF consumption for overweight and obesity. Moreover, the models were conducted to estimate associations between BMI measurements and birth weight and risk ratios of birth weight for overweight and obesity, stratified according to the quartiles of UPF consumption. Two models were conducted: the crude model was unadjusted; in model 2, we adjusted for children’s age at assessment, sex, physical activity, total daily energy intake, delivery mode, breastfeeding, maternal education, maternal BMI status, gestational age at birth and annual family income. Crude and fully adjusted coefficients for independent variables, odds ratios (ORs) for overweight and obesity and 95% confidence intervals (CIs) were derived from the models. The median value of UPF consumption was assigned to each quartile and treated as a continuous variable for linear trend tests. To evaluate the effects of modifications in UPF consumption and the effects of interactions between birth weight and UPF consumption on BMI, BMI z-score or the risk of overweight and obesity were tested by including the interaction terms in the models. Stratified analyses were conducted, comparing children with low or high birthweight with the NBW children within quartiles of UPF consumption in generalized linear models after adjustment for all confounders. Further, we estimated the joint effect of birth weight on BMI values, where stratified analyses were conducted, comparing children in other levels of UPF consumption with normal, low and high birthweight with the NBW children in Q1. The models without any adjustment (crude) were conducted, and then were adjusted for children’s age at assessment, sex, physical activity, total daily energy intake, delivery mode, breastfeeding, maternal education, maternal BMI, gestational age at delivery and annual family income.

We also carried out sensitivity analyses by categorizing UPF consumption into four quartiles, expressed as a percentage of weight contribution toward daily food intake, to determine whether this had any impact on the results. Statistical significance for all data was set at 0.05. All analysis was carried out in IBM SPSS Statistics, version 26.0 (IBM Corporation, New York, NY, USA) and R for Mac, version 4.0.3.

## 3. Results

### 3.1. Participant Characteristics

Of the 1370 children (mean age [SD] = 10.7 [2.6] years) included in this study, 727 [53.1%] were male and 643 [46.9%] were female (Table 1). The characteristics of the children and their mothers are summarized in Table 1. Of all the children, 76.3% (*n* = 1045) were born with NBW and 2.3% (*n* = 32) and 21.4% (*n* = 293) were LBW and HBW, respectively. The mean percentage (SD) of daily energy intake from UPF was 27.7% (12.3%), and that by quartiles was 12.5% (0.2%) in Q1 (lowest), 24.6% (0.2%) in Q2, 33.1% (0.1%) in Q3 and 43.7% (0.4%) in Q4 (highest) (Table 1). The groups, by quartile, had similar child birth weight, gestational age at delivery, breastfeeding and maternal BMI status, but differed in children’s age, sex, delivery mode, physical activity, maternal education, and family income (Table 1). The children’s body weight, BMI and BMI z-score significantly increased with UPF consumption, expressed as the percentage of energy intake from UPF (all *p* < 0.050). Using WGOC criteria, the prevalence of underweight, overweight and obesity among children were, respectively, 9.1%, 10.8% and 8.7%. The childhood BMI status was different within four quartiles (*p* = 0.006).

### 3.2. UPF Consumption and Daily Nutrient Profiles

The UPF subgroups that contributed to the largest percentage of daily energy were industrial grain foods (10.4%), followed by dairy foods and products (5.0%) and ready-to-eat/heat foods (4.6%) (Supplemental Figure S1). The highest percentage of daily energy was provided by carbohydrates (57.4) in children in the quartile of the lowest energy intake from UPF (Q1), compared with those in other quartiles (*p* < 0.001). Children in Q4 had the highest percentages of daily calories provided by both of fat (30.0%) and protein (16.4%) (all *p* < 0.001, Table 2).

### 3.3. Associations of Birth Weight and Childhood BMI Measurements

HBW children likely had a higher mean BMI value (unadjusted β, 1.52; 95%CI, 1.02–2.01; *p* < 0.001) and BMI z-score (unadjusted β, 0.41; 95%, 0.22–0.60, *p* < 0.001) compared with those with NBW (Table 3). HBW was also significantly associated with the risk of overweight and obesity (unadjusted OR, 1.90; 95%CI, 1.38–2.62; *p* < 0.001) (Table 3). The associations remained significant in BMI and BMI z-score after adjusting for potential confounding effects from the child’s age, sex, physical activity, total daily energy intake, breastfeeding, delivery mode, gestational age at delivery, maternal education, maternal BMI status and family income (all *p* < 0.050).

### 3.4. Associations of UPF Consumption and Childhood BMI Measurements

The greater BMI and BMI z-score became insignificant in children with higher energy intake from UPF (Q3 and Q4) after the adjustment for covariates, compared with those with the lowest energy intake from UPF (Q1) (Table 3). No significant association between UPF consumption, expressed as the percentage of energy intake from UPF, and the risk of overweight or obesity was found in the unadjusted or adjusted models (Table 3).

### 3.5. Birthweight and BMI Outcomes by Quartiles of UPF Consumption

There were significant interactions between UPF consumption and birth weight with the children’s BMI and BMI z-score (interaction *p* ≤ 0.001; Table 4). A U-shaped relationship between birth weight and BMI measurements was found after adjusting for potential confounding factors in the presence of interaction terms (all *p* ≤ 0.001). However, birth weight was not significantly associated with the risk of overweight and obesity (*p* > 0.050) (Table 4).

The subgroup analyses stratified by quartiles of UPF consumption expressed as the percentage of energy intake from UPF were performed in Table 4. After adjusting for the confounding variables mentioned above, a U-shaped relationship between birth weight and BMI and BMI z-score was found among children in the lowest quartile of energy intake from UPF (Q1), while a J-shaped relationship occurred in children with the highest quartile of energy intake from UPF (Q4) (all *p* < 0.050). Compared with participants who had normal birth weight, children with HBW tended to have a significant higher BMI and BMI z-score in the lowest quartile (Q1) (BMI: adjusted β = 1.80, 95%CI 0.59–3.00, *p* = 0.001; BMI z-score: adjusted β = 0.71, 95%CI 0.29–1.13, *p* = 0.004) and in the highest quartile (Q4) (BMI: adjusted β = 1.65, 95%CI 0.50–2.79, *p* = 0.005; BMI z-score: adjusted β = 0.63, 95%CI 0.21–1.04, *p* = 0.003) (Table 4). Higher BMI (adjusted β = 6.30; 95%CI 1.75–10.85; *p* = 0.007) and BMI z-score (adjusted β = 2.30; 95%CI 0.72–2.89; *p* = 0.004) among LBW children were only found at the lowest energy intake in the UPF group (Q1) (Table 4). Birth weight was not significantly associated with the risk of overweight and obesity among children in all of four quartiles (all *p* > 0.050) (Table 4).

**Figure 2:**
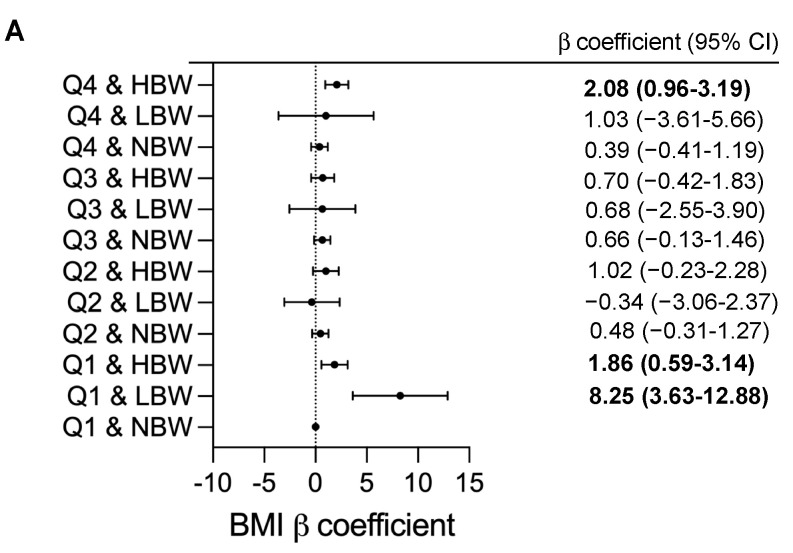

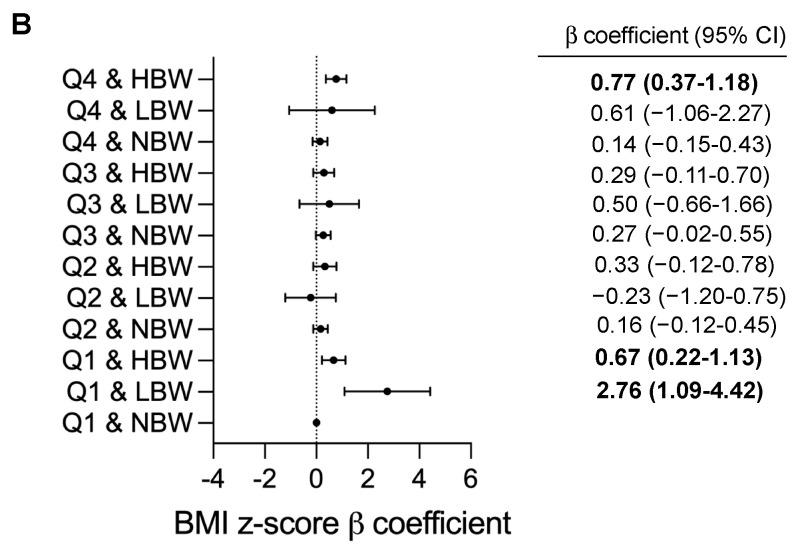
Adjusted β coefficients (95%CI) for childhood BMI measurements according to combined effect of birthweight and UPF consumption. BMI, body mass index; CI, confidence interval; Q: quartile; NBW, normal birthweight; LBW, low birthweight; HBW, high birthweight. β coefficients (95%CI) were calculated using a generalized linear model and adjusted for child’s age and sex (male or female), family income (annual incomes <80,000 RMB, 80,000 to 150.000 RMB or ≥150,000 RMB); maternal education (less than high school, high school and general equivalency, college graduate and above), maternal BMI status (<18.5, 18.5 to 24, 24 to 28, ≥28), physical activity (moderate-to-vigorous physical activity per day <30 min, 30 to 60 min or ≥60 min), child’s total daily energy intake (continuous), breastfeeding (Never, <6 month, ≥6 months), gestational age at delivery (Preterm and early term, full term, late or post-term) and delivery mode (vaginal delivery, caesarean delivery). Q1 and NBW as reference; *p*-value < 0.05 are in bold type. (**A**) Adjusted β coefficients (95%CI) for childhood BMI, (**B**) Adjusted β coefficients (95%CI) for childhood BMI z-score.

We further investigated the joint effects of birth weight and UPF consumption on BMI and BMI z-score (Figure 2). Joint associations between birthweight and UPF consumption were significantly found with children’s BMI and BMI z-score (*p* = 0.001). Compared with children with NBW in the lowest quartile, the BMI value was significantly higher in subgroups of children with LBW in Q1 (adjusted β = 8.25, 95%CI 3.63–12.88, *p* < 0.001), those with HBW in Q1 (adjusted β = 1.86, 95%CI 0.59–3.14, *p* = 0.004) and in Q4 (adjusted β = 2.08, 95%CI 0.96–3.19, *p* < 0.001) (Figure 2A). In addition, the greater BMI z-score were found in subgroups of children with LBW in Q1 (adjusted β = 2.76, 95%CI 1.09–4.42, *p* = 0.001), those with HBW in Q1 (adjusted β = 0.67, 95%CI 0.22–1.13, *p* = 0.004) and in Q4 (adjusted β = 0.77, 95%CI 0.37–1.18, *p* < 0.001) (Figure 2B). After performing sensitivity analyses, the findings were similar to the main outcomes and would lead to similar conclusions about the modified effect of UPF consumption and birthweight on childhood BMI values (Supplemental Table S2 and Figure S2).

## 4. Discussion

In this retrospective cohort study of schoolchildren from six cities across eastern China, we examined the associations of birth weight and UPF consumption with body composition and the interaction between birth weight and UPF consumption for the prediction of childhood BMI measures and overweight/obesity. The results showed that birth weight was positively associated with BMI and BMI for age z-score in childhood, and HBW children had greater BMI values compared with those with NBW after adjustment for total energy intake and other potential confounding factors. Importantly, UPF consumption appears to modify the association with birth weight, especially for LBW. The associations between HBW and LBW and BMI and BMI z-score were found among children with the lowest energy intake from UPF (Q1), whereas only HBW was associated with greater BMI measures in those with the highest energy intake from UPF (Q4). The combination of birth weight and childhood UPF consumption had an additive effect on the increase in BMI and BMI for age z-score. Our findings support the hypothesis that UPF consumption could be a mediator for the association of birth weight with childhood growth.

Food processing alters food structure, content and bioavailability, leading to changes in health outcomes throughout the life course [42]. Growing consumption worldwide has directed attention to UPF as an underlying driver of the obesity epidemic owing to their poorer nutrient profile [43,44]. Our results show that the UPF subgroups industrial grain foods and the ready-to-heat/eat products contributed, respectively, to the largest and the third-most percentage of energy intake among children, which is in accordance with findings by Wang et al. [13]. However, there is an inconsistent finding on the second-most energy source, which was sweet snacks and sweets by 12.9% among US children [13], but in our study it was the dairy foods and products for Chinese children (5.0%). This might be attributed to the food availability and accessibility on the market potentially promoting UPF consumption by children, which varied depended on income growth, urbanization and demographics [43,45,46].

It is known that the addition of UPF to the diet tends to increase daily energy intake with excessive content of fat, free sugar, sodium and low dietary fiber [10,11]. Rauber et al. reported an increased energy intake from carbohydrates and total fats as UPF consumption raised [47]. Similarly in our study, children in Q4 obtained the highest daily energy intake from fats compared to those in other quartiles. Unexpectedly, the daily energy intake from carbohydrates decreased with the addition of UPF. This could be explained by the highest grain intake being in Q1 (33.3% of daily energy), which was four-fold higher than those in Q4. The grains (unprocessed food) in our study, consisting of rice, sticky rice, porridge, home-made noodles and steamed bread, are starchy foods and the main source of carbohydrates in the traditional Chinese diet. Therefore, our results show a different picture from the previous study: energy intake from carbohydrates was reduced, while that from total fats was increased as UPF consumption rose among Chinese children.

A number of studies support that a greater intake of UPF is associated with overweight or obesity [17,18,19,20,21,22,23]. However, in our study, UPF consumption was not significantly associated with BMI values or the risk of overweight and obesity among children after the adjustment for confounding factors, which is in accordance with a prior cross-sectional study based on the UK National Diet and Nutrition Survey [48] and follow-up studies in Brazil and Portugal [49,50]. The mean percentage of energy provided by UPF consumption among our school-aged children was 27.7%, which was similar to Portuguese schoolchildren (29.3%) [50] but obviously lower than those in US (66.2%), UK (65.4%) and Brazil (47.8%) [13,14,49]. Therefore, it is supposed that the low level of UPF consumption might have limited effects on childhood BMI values and the risk of overweight and obesity.

Several observational studies have described positive associations between birth weight and BMI or the risk of childhood obesity [17,24,25,26,27,51]. An international cross-sectional study from 32 countries also reported a positive linear relationship between birth weight and BMI in children aged 6–7 years [18]. Similarly, our result showed that birthweight was a predictor of increased BMI z-score and BMI. This finding was also seen by the addition of the interaction term. Further, our results provide consistent evidence that HBW is a permanent risk factor for childhood BMI values compared with NBW.

Some previous studies demonstrated a J-shaped or U-shaped relationship for LBW and HBW with BMI or the risk of overweight and obesity [18,19,21,32]. In particular, the relationships between LBW and the makers of body weight appear to be controversial. One study [19] found a positive association between LBW and BMI for age z-score only in boys aged 9–11 years, whereas four studies [28,29,31,32] reported that LBW was associated with a decreased effect on the development of overweight and obesity. Others [18,21] showed no significant associations. In agreement with these findings, our subgroup analysis revealed that LBW was associated with a high risk of high BMI and BMI z-score among children in Q1 (the lowest consumption of UPF) but had no association with those in other quartiles, implicating some factors contributing to the observed relation. Previous studies demonstrated that LBW does not directly lead to the occurrence of obesity and instead results in excessive weight gain in the early years of life, which is associated with the increased BMI in childhood [51,52,53]. A prior randomized clinical trial reported that a long-term high-carbohydrate diet could be harmful for anthropometric profiles among obese children [54]. As mentioned above, the dietary intake of carbohydrates was the highest among children in Q1. In addition, no association between LBW and BMI values was found among children in other quartiles. Therefore, our findings possibly indicate that the association of LBW with BMI measures is sensitive to a higher consumption of carbohydrates. Moreover, the association between HBW and BMI values was found in children in Q4 who had the highest daily energy from fats compared to the others. Taken together, nutrient profile, in particular carbohydrates or fat content in diet, might be an important factor to influence the association between birth weight and markers of body composition among children, which is needed for further investigations. Previous studies have investigated the fact that the relationship between birth weight and BMI is confounded by factors such as maternal weight or BMI, gestation age, infant growth and socioeconomic status [51,55]. To our knowledge, we are the first researchers to investigate dietary intake as a mediator for the relationships between birth weight and markers of body weight among children. Thus, our findings are important because they provide an initial step to a better understanding of dietary factors and the inconsistent association between birth weight and adiposity outcomes among children.

The main strengths of this study lie in a relatively large sample size and the generalization of the findings to a larger population because of the recruitment of a multicity sample with low- to high-socioeconomic status, providing insights into dietary factors moderating the association between birth weight and adiposity that can guide future prospective studies.

Our study has several limitations. First, this study was a retrospective cohort design, which limits the ability to assess body weight at a one-time point, so we could not capture changes in body weight over time. Second, we obtained information on dietary intake performed with the FFQ that may under- or overestimate total energy intake and was not specifically used to collect data focusing on food classification, which probably limits out=r ability to accurately capture UPF consumption. However, the data collected by FFQ have been considered a useful tool for assessing relative intake in the context of a higher versus lower level of UPF intake over time [56,57]. In addition, UPF consumption as a percentage of total energy intake in our study could minimize under- or overestimation bias [57]. Third, misclassifications in the NOVA cannot be ruled out. However, this would have led to a nondifferential measurement error, if present, potentially biasing results towards the null hypothesis [46]. Fourth, residual confounding cannot be entirely ruled out from this study. Although we adjusted for several major variables in our models, the possibility of residual bias remains, as we may not have been able to fully control for potential unmeasured or unknown factors, such as maternal pre-pregnancy BMI, that were not available in this study. Instead, we adjusted for maternal BMI status. Fifth, the effects of a smaller number of LBW children on the results is uncertain. Nevertheless, it is important to keep in mind that several previous findings were in line with the plausibility of these findings, and the results remained unchanged after a sensitivity analysis adjusting for many confounders.

## 5. Conclusions

This study allowed us to observe the associations between birth weight and increased BMI and BMI z-score among Chinese children. We also report for the first time that, to the best of our knowledge, UPF consumption may modify the relationship between birth weight and markers of adiposity, especially LBW. Our findings also suggest that the modification effect of nutrient profile, particularly carbohydrates, may be a true outcome of low birth weight that caregivers and health professionals should weigh when promoting healthy eating behaviors regarding weight problems among children. Future research is still needed with more adequate, high-quality and prospective data to validate these findings.

## Figures and Tables

**Table 1:** Sociodemographic characteristics by quartiles of UPF consumption among children.

		Percentage of Energy Intake from UPF				
Characteristics	Overall (*n* = 1370)	Q1 (*n* = 374)	Q2 (*n* = 369)	Q3 (*n* = 308)	Q4 (*n* = 317)	*p* Value ^e^
Mean (SD) [range], % ^a^	27.7 (12.3) [0.0–73.8]	12.5 (4.7) [0.0–19.2]	24.6 (3.0) [19.3–29.3]	33.1 (2.2) [19.3–36.7]	43.7 (0.4) [36.8–73.8]	**0.000**
Total energy intake, kcal	2804.6 (35.3)	2891.5 (64.2)	2915.7 (71.1)	2732.9 (75.9)	2634.1 (70.8)	**0.000**
Age, y	10.7 (2.6)	10.2 (2.7)	10.6 (2.7)	10.9 (2.4)	11.1 (0.1)	**0.001**
Sex		374	369	308	317	**0.038**
Male	727 (53.1)	177 (47.3)	197 (53.4)	179 (58.1)	174 (54.5)	
Female	643 (46.9)	197 (52.7)	172 (46.6)	129 (41.9)	145 (45.5)	
Weight, kg	39.4 (14.6)	36.6 (14.6)	38.2 (14.3)	41.2 (14.3)	42.6 (14.5)	**0.000**
Height, cm	145.7 (17.2)	142.5 (18.3)	144.9 (16.9)	147.1 (16.8)	149.3 (15.6)	**0.000**
BMI	18.0 (3.7)	17.3 (3.5)	17.7 (3.7)	18.5 (3.7)	18.7 (3.8)	**0.000**
BMI z-score	0.2 (1.4)	0.0 (1.2)	0.1 (1.6)	0.4 (1.3)	0.4 (1.3)	**0.000**
BMI status		347	334	277	278	**0.006**
Underweight	112 (9.1)	40 (11.5)	29 (8.7)	27 (9.7)	16 (5.8)	
Normal	882 (71.4)	250 (72.0)	247 (74.0)	181 (197.7)	204 (73.4)	
Overweight	134 (10.8)	40 (11.5)	33 (9.9)	36 (30.0)	25 (9.0)	
Obese	108 (8.7)	17 (4.9)	25 (7.5)	33 (24.2)	33 (11.9)	
Birthweight ^b^						0.062
LBW	32 (2.3)	11 (2.9)	11 (3.0)	5 (1.6)	5 (1.6)	
NBW	1045 (76.3)	291 (77.8)	294 (79.7)	229 (74.4)	231 (72.4)	
HBW	293 (21.4)	72 (19.3)	64 (17.3)	74 (24.0)	83 (26.0)	
Delivery mode		367	353	293	306	**0.025**
Vaginal	546 (41.4)	174 (47.4)	146 (41.4)	106 (36.2)	120 (39.2)	
Caesarean	773 (58.6)	193 (52.6)	207 (58.6)	187 (63.8)	186 (60.8)	
Gestational age ^c^		366	351	292	302	0.939
Pre and early term	60 (4.6)	17 (4.6)	14 (4.0)	12 (4.1)	17 (5.6)	
Full term	1103 (84.1)	308 (84.2)	300 (85.5)	247 (84.6)	248 (82.1)	
Late or post term	148 (11.3)	41 (11.2)	37 (10.5)	33 (11.3)	37 (12.3)	
Duration of Breastfeeding		335	324	277	283	**0.053**
Never	197 (16.2)	44 (13.1)	54 (16.7)	50 (18.1)	49 (17.3)	
<6 months	144 (11.8)	29 (8.7)	45 (13.9)	28 (10.1)	42 (14.8)	
≥6 months	878 (72.0)	262 (78.2)	225 (69.4)	199 (71.8)	192 (67.8)	
Physical activity		219	271	274	287	**0.025**
<30 min	651 (61.9)	126 (57.5)	189 (69.7)	159 (58.0)	177 (61.7)	
30–60 min	273 (26.0)	68 (31.1)	57 (21.0)	71 (25.9)	77 (26.8)	
≥60 min	127 (12.1)	25 (11.4)	25 (9.2)	44 (16.1)	33 (11.5)	
Maternal BMI status		225	275	283	297	0.576
Underweight	66 (6.1)	13 (5.8)	21 (7.6)	16 (5.7)	16 (5.4)	
Normal	698 (64.6)	133 (59.1)	179 (65.1)	187 (66.1)	199 (67.0)	
Overweight	259 (24.0)	66 (29.3)	64 (23.3)	63 (22.3)	66 (22.2)	
Obese	57 (5.3)	13 (5.8)	11 (14.5)	17 (6.0)	16 (5.4)	
Maternal education		363	362	291	296	**0.024**
<High school	488 (37.2)	135 (37.2)	118 (32.6)	122 (41.9)	113 (38.2)	
High school or equivalent	333 (25.4)	75 (20.7)	102 (28.2)	79 (27.1)	77 (26.0)	
College graduate and above	491 (37.4)	153 (42.1)	142 (39.2)	90 (30.9)	106 (35.8)	
Family income ^d^		351	339	275	282	**0.013**
Low	386 (31.0)	108 (30.8)	91 (26.8)	97 (35.3)	90 (31.9)	
Middle	402 (32.2)	93 (26.5)	118 (34.8)	95 (34.5)	96 (34.0)	
High	459 (36.8)	150 (42.7)	130 (38.3)	83 (30.2)	96 (34.0)	

Abbreviations: UPF, ultra-processed foods; Q, quartiles; PA, physical activity; NBW, normal birthweight; LBW, low birthweight; HBW, high birthweight; Pre and early term, preterm and early term. ^a^ Percentage of energy intake from UPF was defined as the proportion of calorie intake from ultra-processed foods relative to daily energy intake and was categorized into quartiles (Q1–Q4 represents lowest to highest quartile of percentage of energy intake from UPF consumption). A set of cutoff points for quartiles of energy from UPF consumption was defined at 19.2%, 29.4% and 36.8% of daily energy intake. ^b^ NBW was defined as birthweight relative to 2500–3999 g; LBW was defined as birthweight less than 2500 g; HBW was defined as birthweight more than 4000 g. ^c^ Preterm and early term was defined as gestational age relative to 20–38 completed weeks; full term was defined as gestational age relative to 39–40 completed weeks; late or post-term was defined as gestational age relative to more than 41 weeks. ^d^ Family income was defined as low relative to less than 80,000 RMB per year, middle relative to 80,000–150,000 RMB and high relative to more than 150,000 RMB. ^e^ Kruskal–Wallis 1-way ANOVA tests were used for continuous variables, and χ^2^ tests or Fisher’s exact tests were used for categorical variables to examine differences in characteristics between the quartiles of UPF consumption. *p*-value < 0.05 are in bold type.

**Table 2:** Nutrient profile by quartiles of UPF consumption among children.

Nutrient Profiles	Percentage of Energy Intake from UPF ^a^					
	Overall Mean (SD)	Q1 Mean (SD)	Q2 Mean (SD)	Q3 Mean (SD)	Q4 Mean (SD)	*p* Value ^b^
Total energy intake (kcal)	2804.6 (35.3)	2891.5 (64.2)	2915.7 (71.1)	2732.9 (75.9)	2634.1 (70.8)	**<0.001**
Total protein, % of energy	15.9 (3.2)	14.7 (0.2)	15.7 (0.1)	16.4 (0.2)	16.4 (0.2)	**<0.001**
Total fat, % of energy	26.6 (7.2)	22.5 (0.4)	25.0 (0.3)	28.0 (0.3)	30.0 (0.3)	**<0.001**
Total carbohydrate, % of energy	53.1 (9.2)	57.4 (0.6)	54.4 (0.4)	51.2 (0.4)	50.1 (0.4)	**<0.001**
Unprocessed foods, % of energy	63.3 (0.4)	81.6 (0.4)	66.1 (0.4)	55.6 (0.4)	45.9 (0.4)	**<0.001**
Grains	19.8 (0.4)	33.3 (0.9)	22.4 (0.6)	12.8 (0.5)	7.8 (0.4)	**<0.001**
Meat, Poultry, Fish and Eggs	16.5 (0.2)	19.6 (0.6)	16.6 (0.5)	15.5 (0.4)	13.8 (0.4)	**<0.001**
Milk and plain yogurt	1.4 (0.1)	2.2 (0.1)	1.4 (0.1)	0.9 (0.1)	0.8 (0.1)	**<0.001**
Fruits	6.7 (0.1)	8.5 (0.3)	6.9 (0.2)	6.0 (0.2)	5.1 (0.2)	**<0.001**
Vegetables	6.9 (0.1)	7.5 (0.4)	6.7 (0.3)	6.9 (0.2)	6.3 (0.2)	**0.016**
Mushroom and Alga	0.3 (0.0)	0.3 (0.0)	0.2 (0.0)	0.3 (0.0)	0.3 (0.0)	**<0.001**
Nuts, Seeds and Legumes	4.4 (0.1)	3.2 (0.2)	4.5 (0.2)	5.4 (0.2)	4.9 (0.2)	**<0.001**
Others	10.3 (0.2)	9.6 (0.4)	10.5 (0.3)	11.5 (0.3)	9.7 (0.3)	**<0.001**

^a^ Percentage of daily energy intake from UPF was defined as the proportion of calorie intake from ultra-processed foods relative to daily energy intake and was categorized into quartiles (Q1–Q4 represents lowest to highest quartile of percentage of energy intake from UPF consumption). ^b^ Kruskal–Wallis 1-way ANOVA tests were used to examine differences between the quartiles of UPF consumption. *p*-value < 0.05 are in bold type.

**Table 3:** Associations of birthweight and UPF consumption with BMI, BMI z-score and risk of overweight and obesity among children in unadjusted models and adjusted models without interaction.

			BMI		BMI z-Score		Risk of Overweight or Obesity	
			β Coefficient (95% CI)	*p*-Value ^b^	β Coefficient (95% CI)	*p*-Value ^b^	OR (95% CI)	*p*-Value ^b^
Birthweight								
	unadjusted model	NBW	Ref.	--	Ref.	--	Ref.	--
		LBW	1.06 (−0.24 to 2.36)	0.109	0.48 (−0.01 to 0.97)	0.055	1.77 (0.78 to 4.05)	0.175
		HBW	1.52 (1.02 to 2.01)	**<0.001**	0.41 (0.22 to 0.60)	**<0.001**	1.90 (1.38 to 2.62)	**<0.001**
		*p*-value ^b^	**<0.001**		**<0.001**		**<0.001**	
	adjusted model	NBW	Ref.	--	Ref.	--	Ref.	--
		LBW	1.02 (−0.84 to 2.89)	0.283	0.40 (−0.27 to 1.07)	0.246	0.98 (0.22 to 4.25)	0.973
		HBW	0.99 (0.39 to 1.58)	**0.001**	0.36 (0.15 to 0.58)	**0.001**	1.22 (0.79 to 1.88)	0.382
		*p*-value ^b^	**0.003**		**0.003**		0.679	
UPF Consumption ^a^								
	unadjusted model	Q1	Ref.		Ref.		Ref.	
		Q2	0.41 (−0.13 to 0.94)	0.134	0.04 (−0.16 to 0.24)	0.699	0.91 (0.66 to 1.25)	0.569
		Q3	1.07 (0.52 to 1.63)	**<0.001**	0.31 (0.10 to 0.52)	**0.003**	1.30 (0.94 to 1.79)	0.115
		Q4	1.37 (0.83 to 1.92)	**<0.001**	0.35 (0.15 to 0.55)	**0.001**	0.95 (0.69 to 1.32)	0.758
		*p*-value for trend ^b^	**<0.001**		**<0.001**		0.164	
	adjusted model	Q1	Ref.	--	Ref.	--	Ref.	--
		Q2	0.07 (−0.65 to 0.78)	0.859	0.01 (−0.25 to 0.26)	0.961	1.01 (0.57 to 1.80)	0.976
		Q3	0.15 (−0.56 to 0.86)	0.675	0.09 (−0.16 to 0.35)	0.481	1.27 (0.73 to 2.22)	0.395
		Q4	0.29 (−0.42 to 1.00)	0.421	0.11 (−0.14 to 0.37)	0.386	1.03 (0.58 to 1.82)	0.927
		*p*-value for trend ^b^	0.861		0.746		0.763	

Abbreviations: BMI, body mass index; CI, confidence interval; UPF: ultra-processed foods, Q: quartiles; NBW, normal birthweight; LBW, low birthweight; HBW, high birthweight. ^a^ A set of cutoff points for quartiles of energy from UPF consumption was defined at 19.2%, 29.4% and 36.8% of daily energy intake. ^b^ Generalized linear models were used to investigate associations of birthweight and UPF consumption with BMI measures in unadjusted models and were further adjusted for the child’s age and sex (male or female), family income (annual incomes <80,000 RMB, 80,000 to 150,000 RMB or ≥150,000 RMB), maternal education (less than high school, high school and general equivalency, college graduate and above), maternal BMI status (<18.5, 18.5 to 24, 24 to 28, ≥28), physical activity (moderate-to-vigorous physical activity per day <30 min, 30 to 60 min or ≥60 min), child’s total daily energy intake (continuous), breastfeeding (Never, <6 month, ≥6 months), gestational age at delivery (Preterm and early term, full term, late or post-term) and delivery mode (vaginal delivery, caesarean delivery). NBW and Q1 as reference; *p*-value < 0.05 are in bold type.

**Table 4:** Adjusted β coefficient or OR (95%CI) for childhood adiposity in relation to birthweight by quartiles of UPF consumption.

Birthweight		UPF Consumption ^a^										
		Overall		Q1		Q2		Q3		Q4		*p* for Interaction ^b^
		β Coefficient (95% CI)	*p*-Value ^b^	β Coefficient (95% CI)	*p*-Value ^b^	β Coefficient (95% CI)	*p*-Value ^b^	β Coefficient (95% CI)	*p*-Value ^b^	β Coefficient (95% CI)	*p*-Value ^b^	
BMI												**0.006**
	NBW	Ref.	--	Ref.	--	Ref.	--	Ref.	--	Ref.	--	
	HBW	1.86 (0.59 to 3.14)	**0.004**	1.80 (0.59 to 3.00)	**0.004**	0.59 (−0.74 to 1.93)	0.385	0.20 (−0.85 to 1.25)	0.146	1.65 (0.50 to 2.79)	**0.005**	
	LBW	8.25 (3.63 to 12.88)	**0.000**	6.30 (1.75 to 10.85)	**0.007**	−0.63 (−3.56 to 2.30)	0.673	1.86 (−2.28 to 6.00)	0.378	1.94 (−2.78 to 6.66)	0.421	
	*p* value ^b^	**0.001**		**0.001**		0.607		0.658		**0.015**		
BMI z-score												**0.009**
	NBW	Ref.	--	Ref.	--	Ref.	--	Ref.	--	Ref.	--	
	HBW	0.67 (0.22 to 1.13)	**0.004**	0.71 (0.29 to 1.13)	**0.001**	0.21 (−0.25 to 0.68)	0.370	0.07 (−0.33 to 0.47)	0.726	0.63 (0.21 to 1.04)	**0.003**	
	LBW	2.76 (1.09 to 4.42)	**0.001**	2.30 (0.72 to 2.89)	**0.004**	−0.30 (−1.32 to 0.73)	0.569	0.77 (−0.79 to 2.34)	0.332	0.81 (−0.92 to 2.53)	0.358	
	*p* value ^b^	**0.000**		**0.000**		0.544		0.610		**0.010**		
Risk of overweight or obesity		OR (95% CI)		OR (95% CI)		OR (95% CI)		OR (95% CI)		OR (95% CI)		**0.369**
	NBW	Ref.	--	Ref.	--	Ref.	--	Ref.	--	Ref.	--	
	HBW	2.38 (0.91 to 6.21)	0.076	2.01 (0.69 to 5.85)	0.200	0.95 (0.32 to 2.79)	0.926	0.72 (0.30 to 1.69)	0.445	2.01 (0.81 to 4.96)	0.132	
	LBW	4.91 (0.26 to 92.78)	0.288	3.35 (0.09 to 122.06)	0.510	1.08 (0.08 to 14.77)	0.955	0.64 (0.02 to 18.84)	0.794	NA	NA	
	*p* value ^b^	0.619		0.383		0.994		0.735		0.321		

Abbreviations: BMI, body mass index; CI, confidence interval; UPF, ultra-processed foods, Q: quartiles; NBW, normal birthweight; LBW, low birthweight; HBW, high birthweight. ^a^ A set of cutoff points for quartiles of energy from UPF consumption was defined at 19.2%, 29.4% and 36.8% of daily energy intake. Q1 and Q4 indicate the lowest and highest UPF consumption, respectively. ^b^ Model was adjusted for children’s age and sex (male or female), family income (annual incomes <80,000 RMB, 80,000 to 150,000 RMB or ≥150,000 RMB); maternal education (less than high school, high school and general equivalency, college graduate and above), maternal BMI status (<18.5, 18.5 to 24, 24 to 28, ≥28), physical activity (moderate-to-vigorous physical activity per day <30 min, 30 to 60 min, or ≥60 min), children’s total daily energy intake (continuous), breastfeeding (Never, <6 month, ≥ 6 months), gestational age at delivery (Preterm and early term, full term, late or post-term) and delivery mode (vaginal delivery, caesarean delivery). NBW as reference; NA as no applicable; *p*-value < 0.05 are in bold type.

## Data Availability

The datasets used and analyzed in the current study will be made available upon reasonable request.

## Supplementary Materials

The following supporting information can be downloaded at https://www.mdpi.com/article/10.3390/nu15194178/s1, Table S1. Subgroups of UPF and unprocessed foods (NPF) and example foods. Table S2. Results of sensitivity analyses for association of birthweight, UPF consumption with BMI, BMI z-score and risk of overweight and obesity among children in unadjusted models and adjusted models without interaction. Figure S1. Contributions of food groups to UPF consumption among children. Figure S2. Adjusted β coefficient (95%CI) for childhood BMI measurements according to combined effect of birthweight and UPF consumption.

## References

[1] Gungor N.K. (2014). Overweight and obesity in children and adolescents. JCRPE.

[2] Evensen E., Wilsgaard T., Furberg A.S., Skeie G. (2016). Tracking of overweight and obesity from early childhood to adolescence in a population-based cohort—The Tromsø Study, Fit Futures. BMC Pediatr..

[3] Han J.C., Lawlor D.A., Kimm S.Y. (2010). Childhood obesity. Lancet.

[4] Pan X.-F., Wang L., Pan A. (2021). Epidemiology and determinants of obesity in China. Lancet Diabetes Endocrinol..

[5] World Health Organization (2018). Taking Action on Childhood Obesity.

[6] Cecchini M., Sassi F., Lauer J.A., Lee Y.Y., Guajardo-Barron V., Chisholm D. (2010). Tackling of unhealthy diets, physical inactivity, and obesity: Health effects and cost-effectiveness. Lancet.

[7] Mozaffarian D. (2020). Dietary and policy priorities to reduce the global crises of obesity and diabetes. Nat. Food.

[8] Monteiro C.A., Cannon G., Levy R.B., Moubarac J.-C., Louzada M.L.C., Rauber F., Khandpur N., Cediel G., Neri D., Martinez-Steele E. (2019). Ultra-processed foods: What they are and how to identify them. Public Health Nutr..

[9] Monteiro C.A., Cannon G., Moubarac J.C., Levy R.B., Louzada M.L.C., Jaime P.C. (2018). The UN Decade of Nutrition, the NOVA food classification and the trouble with ultra-processing. Public Health Nutr..

[10] Luiten C.M., Steenhuis I.H., Eyles H., Ni Mhurchu C., Waterlander W.E. (2016). Ultra-processed foods have the worst nutrient profile, yet they are the most available packaged products in a sample of New Zealand supermarkets—CORRIGENDUM. Public Health Nutr..

[11] Moubarac J.C., Martins A.P., Claro R.M., Levy R.B., Cannon G., Monteiro C.A. (2013). Consumption of ultra-processed foods and likely impact on human health. Evidence from Canada. Public Health Nutr..

[12] Meyer K.A., Taillie L.S. (2021). Intake of Ultraprocessed Foods Among US Youths: Health Concerns and Opportunities for Research and Policy. JAMA.

[13] Wang L., Martínez Steele E., Du M., Pomeranz J.L., O’Connor L.E., Herrick K.A., Luo H., Zhang X., Mozaffarian D., Zhang F.F. (2021). Trends in Consumption of Ultraprocessed Foods Among US Youths Aged 2–19 Years, 1999–2018. JAMA.

[14] Onita B.M., Azeredo C.M., Jaime P.C., Levy R.B., Rauber F. (2021). Eating context and its association with ultra-processed food consumption by British children. Appetite.

[15] An M., Liu X., Guo H., Zhou Q. (2022). The Associations between Caregivers’ Emotional and Instrumental Feeding, Children’s Emotional Eating, and Children’s Consumption of Ultra-Processed Foods in China. Int. J. Environ. Res. Public Health.

[16] Zhang S., Gan S., Zhang Q., Liu L., Meng G., Yao Z., Wu H., Gu Y., Wang Y., Zhang T. (2021). Ultra-processed food consumption and the risk of non-alcoholic fatty liver disease in the Tianjin Chronic Low-grade Systemic Inflammation and Health Cohort Study. Int. J. Epidemiol..

[17] Fonseca M.J., Severo M., Correia S., Santos A.C. (2015). Effect of birth weight and weight change during the first 96 h of life on childhood body composition—Path analysis. Int. J. Obes..

[18] Mitchell E.A., Stewart A.W., Braithwaite I., Hancox R.J., Murphy R., Wall C., Beasley R., ISAAC Phase Three Study Group (2017). Birth weight and subsequent body mass index in children: An international cross-sectional study. Pediatr. Obes..

[19] Qiao Y., Ma J., Wang Y., Li W., Katzmarzyk P.T., Chaput J.P., Fogelholm M., Johnson W.D., Kuriyan R., Kurpad A. (2015). Birth weight and childhood obesity: A 12-country study. Int. J. Obes. Suppl..

[20] Kapral N., Miller S.E., Scharf R.J., Gurka M.J., DeBoer M.D. (2018). Associations between birthweight and overweight and obesity in school-age children. Pediatr. Obes..

[21] Rugholm S., Baker J.L., Olsen L.W., Schack-Nielsen L., Bua J., Sørensen T.I. (2005). Stability of the association between birth weight and childhood overweight during the development of the obesity epidemic. Obes. Res..

[22] Suchomlinov A., Tutkuviene J. (2014). The relationship between birth weight, adiposity rebound and overweight at the age of 17 years (results of the Lithuanian longitudinal growth study, 1990–2008). Anthropol. Anz..

[23] Reilly J.J., Armstrong J., Dorosty A.R., Emmett P.M., Ness A., Rogers I., Steer C., Sherriff A. (2005). Early life risk factors for obesity in childhood: Cohort study. BMJ.

[24] Brophy S., Cooksey R., Gravenor M.B., Mistry R., Thomas N., Lyons R.A., Williams R. (2009). Risk factors for childhood obesity at age 5: Analysis of the millennium cohort study. BMC Public Health.

[25] Heppe D.H.M., Kiefte-de Jong J.C., Durmuş B., Moll H.A., Raat H., Hofman A., Jaddoe V.W.V. (2013). Parental, fetal, and infant risk factors for preschool overweight: The Generation R Study. Pediatr. Res..

[26] Janjua N.Z., Mahmood B., Islam M.A., Goldenberg R.L. (2012). Maternal and Early Childhood Risk Factors for Overweight and Obesity among Low-Income Predominantly Black Children at Age Five Years: A Prospective Cohort Study. J. Obes..

[27] Johnsson I.W., Haglund B., Ahlsson F., Gustafsson J. (2015). A high birth weight is associated with increased risk of type 2 diabetes and obesity. Pediatr. Obes..

[28] Frye C., Heinrich J. (2003). Trends and predictors of overweight and obesity in East German children. Int. J. Obes. Relat. Metab. Disord..

[29] Hirschler V., Bugna J., Roque M., Gilligan T., Gonzalez C. (2008). Does Low Birth Weight Predict Obesity/Overweight and Metabolic Syndrome in Elementary School Children?. Arch. Med. Res..

[30] Yu Z.B., Han S.P., Zhu G.Z., Zhu C., Wang X.J., Cao X.G., Guo X.R. (2011). Birth weight and subsequent risk of obesity: A systematic review and meta-analysis. Obes. Rev. Off. J. Int. Assoc. Study Obes..

[31] Schellong K., Schulz S., Harder T., Plagemann A. (2012). Birth weight and long-term overweight risk: Systematic review and a meta-analysis including 643,902 persons from 66 studies and 26 countries globally. PLoS ONE.

[32] Oldroyd J., Renzaho A., Skouteris H. (2011). Low and high birth weight as risk factors for obesity among 4 to 5-year-old Australian children: Does gender matter?. Eur. J. Pediatr..

[33] Chen C., Jin Z., Yang Y., Jiang F., Huang H., Liu S., Jin X. (2019). Association of low birth weight with thinness and severe obesity in children aged 3–12 years: A large-scale population-based cross-sectional study in Shanghai, China. BMJ Open.

[34] Costa C.S., Del-Ponte B., Assunção M.C.F., Santos I.S. (2018). Consumption of ultra-processed foods and body fat during childhood and adolescence: A systematic review. Public Health Nutr..

[35] Askari M., Heshmati J., Shahinfar H., Tripathi N., Daneshzad E. (2020). Ultra-processed food and the risk of overweight and obesity: A systematic review and meta-analysis of observational studies. Int. J. Obes..

[36] Cai M., Loy S.L., Tan K.H., Godfrey K.M., Gluckman P.D., Chong Y.S., Shek L.P., Cheung Y.B., Lek N., Lee Y.S. (2018). Association of Elective and Emergency Cesarean Delivery with Early Childhood Overweight at 12 Months of Age. JAMA Netw. Open.

[37] Wada K., Tamakoshi K., Tsunekawa T., Otsuka R., Zhang H., Murata C., Nagasawa N., Matsushita K., Sugiura K., Yatsuya H. (2005). Validity of self-reported height and weight in a Japanese workplace population. Int. J. Obes..

[38] Cole T.J., Bellizzi M.C., Flegal K.M., Dietz W.H. (2000). Establishing a standard definition for child overweight and obesity worldwide: International survey. BMJ.

[39] Li H., Ji C.Y., Zong X.N., Zhang Y.Q. (2009). Body mass index growth curves for Chinese children and adolescents aged 0 to 18 years. Zhonghua Er Ke Za Zhi.

[40] Group of China Obesity Task Force (2004). Body mass index reference norm for screening overweight and obesity in Chinese children and adolescents. Zhonghua Liu Xing Bing Xue Za Zhi.

[41] Ji C.-Y., Working Group on Obesity in China (2005). Report on childhood obesity in China (1)—Body mass index reference for screening overweight and obesity in Chinese school-age children. Biomed. Environ. Sci..

[42] Jones J.M. (2019). Food processing: Criteria for dietary guidance and public health?. Proc. Nutr. Soc..

[43] Baker P., Machado P., Santos T., Sievert K., Backholer K., Hadjikakou M., Russell C., Huse O., Bell C., Scrinis G. (2020). Ultra-processed foods and the nutrition transition: Global, regional and national trends, food systems transformations and political economy drivers. Obes. Rev..

[44] Adams J., Hofman K., Moubarac J.C., Thow A.M. (2020). Public health response to ultra-processed food and drinks. BMJ.

[45] Rauber F., Chang K., Vamos E.P., da Costa Louzada M.L., Monteiro C.A., Millett C., Levy R.B. (2021). Ultra-processed food consumption and risk of obesity: A prospective cohort study of UK Biobank. Eur. J. Nutr..

[46] Beslay M., Srour B., Méjean C., Allès B., Fiolet T., Debras C., Chazelas E., Deschasaux M., Wendeu-Foyet M.G., Hercberg S. (2020). Ultra-processed food intake in association with BMI change and risk of overweight and obesity: A prospective analysis of the French NutriNet-Santé cohort. PLoS Med..

[47] Rauber F., Da Costa Louzada M.L., Steele E.M., Millett C., Monteiro C.A., Levy R.B. (2018). Ultra-Processed Food Consumption and Chronic Non-Communicable Diseases-Related Dietary Nutrient Profile in the UK (2008–2014). Nutrients.

[48] Adams J., White M. (2015). Characterisation of UK diets according to degree of food processing and associations with socio-demographics and obesity: Cross-sectional analysis of UK National Diet and Nutrition Survey (2008–12). Int. J. Behav. Nutr. Phys. Act..

[49] Costa C.S., Rauber F., Leffa P.S., Sangalli C.N., Campagnolo P.D.B., Vitolo M.R. (2019). Ultra-processed food consumption and its effects on anthropometric and glucose profile: A longitudinal study during childhood. Nutr. Metab. Cardiovasc. Dis..

[50] Vedovato G.M., Vilela S., Severo M., Rodrigues S., Lopes C., Oliveira A. (2021). Ultra-processed food consumption, appetitive traits and BMI in children: A prospective study. Br. J. Nutr..

[51] Hui L.L., Schooling C.M., Leung S.S.L., Mak K.H., Ho L.M., Lam T.H., Leung G.M. (2008). Birth Weight, Infant Growth, and Childhood Body Mass Index: Hong Kong’s Children of 1997 Birth Cohort. Arch. Pediatr. Adolesc. Med..

[52] Casey P.H., Bradley R.H., Whiteside-Mansell L., Barrett K., Gossett J.M., Simpson P.M. (2012). Evolution of obesity in a low birth weight cohort. J. Perinatol..

[53] Parsons T.J., Power C., Logan S., Summerbell C.D. (1999). Childhood predictors of adult obesity: A systematic review. Int. J. Obes. Relat. Metab. Disord..

[54] Kirk S., Brehm B., Saelens B.E., Woo J.G., Kissel E., D’Alessio D., Bolling C., Daniels S.R. (2012). Role of Carbohydrate Modification in Weight Management among Obese Children: A Randomized Clinical Trial. J. Pediatr..

[55] Parsons T.J., Power C., Manor O. (2001). Fetal and early life growth and body mass index from birth to early adulthood in 1958 British cohort: Longitudinal study. BMJ.

[56] Nagel G., Zoller D., Ruf T., Rohrmann S., Linseisen J. (2007). Long-term reproducibility of a food-frequency questionnaire and dietary changes in the European Prospective Investigation into Cancer and Nutrition (EPIC)-Heidelberg cohort. Br. J. Nutr..

[57] Steinemann N., Grize L., Ziesemer K., Kauf P., Probst-Hensch N., Brombach C. (2017). Relative validation of a food frequency questionnaire to estimate food intake in an adult population. Food Nutr. Res..

