# Supplementary material for: Ultra-Processed Food as Mediator of the Association between Birthweight and Childhood Body Weight Outcomes: A Retrospective Cohort Study
Source: Nutrients. 2023 Sep 27;15(19):4178. doi: 10.3390/nu15194178 (PMC10574691; doi:10.3390/nu15194178)
Supplement: Supplementary file 1 [file nutrients-15-04178-s001.zip › nutrients-2602849-SI.pdf]

Ultra-Processed Food as Mediator of the Association between Birthweight and Childhood Body Weight Outcomes: A Retrospective Cohort Study

Min Hou [1*], *Chao Qiu* [2*]

[a] School of Public Health, College of Medicine, Shanghai Jiao Tong University, 227 Chongqing South Road, Shanghai 200025, China

[b] College of Humanities, Jiangnan University, 1800 Lihu Road, Wuxi 214122, China

Corresponding authors: Min Hou (e-mail; minhou@sjtu.edu.cn); Chao Qiu (e-mail; Chao.Qiu@outlook.com)

Table S1. Subgroups of UPF and unprocessed foods (NPF) and example foods

**UPF subgroup**	Foods or Beverages included
Industrial grain foods	Deep-fried bread stick, White bread, Whole wheat bread, Cake, Sweet dumplings, Sesame paste, breakfast oatmeal, biscuits
Ready-to-eat and fast foods	Pizza, Hamburger, Fried chicken
Sweet snacks and sweets	Chocolate, Candy, Haw flakes
Meat snacks	Sliced dried beef
Savory snacks	Chips
Sugar sweetened beverages	Sweetened fruit and vegetable juices, Carbonated drinks, Sweetened tea drinks
Dairy foods and products	Sweetened yoghurt, Flavored yogurt, Dried milk with sugar, Soy milk, Chocolate milk, Cream
Fast-food potato products	French fries
Soy products	Dried tofu
Other ultraprocessed foods	Liquid drink, Salted cucumber, Salted vegetables, Fermented Tofu
**NPF subgroup**	
Grains	Rice, Sticky rice, Porridge, Home-made noodles, Steamed bread
Meat, Poultry, fish and eggs	Pork, Beef, Lamb, Pork ribs, Animal liver, Blood products, Chicken, Duck, Goose, Dove, Freshwater fish, Sea fish, Shrimp, Crab, Quail egg, Egg, Duck egg
Milk and plain yogurt	Milk, plain yogurt
Fruits	Orange, Tangerine, Watermelon, Pineapple, Apple, Longan, Pear, Grape, Banana, Hami melon, Kiwi fruit, Strawberry
Vegetables	Spinach, Chinese cabbage, Lettuce, Cauliflower, Broccoli, Purple cabbage, Cabbage, Amaranth, Lettuce, Rapeseed, Leek, Coriander, Celery, Onions, Garlic seedlings, Bean sprouts, Sweet potato, Potato, Lotus root, Garlic sprout, Wax gourd, Chayote, Cucumber, Pumpkin, Loofah, Courgette, Tomato, Pepper (green), Eggplant, Beans, Corn, Carrot, White radish
Mushroom and alga	Lentinula edodes, Oyster mushroom, Nori, Kelp, Fungus
Nuts, seeds, or legumes	Soybean, Peas, Soybean, Mung bean, Red beans, Black beans, Peanut, Chestnut, Sesame
Others	Vegetarian stuffed dumplings, Meat stuffed dumplings, Meat buns, Vegetable buns, Bean curd, Bean blossom tofu, Soy milk, Dried dates, Raisin, Dried persimmon, Green tea, Black tea, Coffee

Table S2. Results of sensitivity analyses for Association of Birthweight, UPF consumption with BMI, BMI z-score and Risk of Overweight and Obesity Among Children in Unadjusted Models and Adjusted Models without interaction

		BMI		BMI z- score		Risk of overweight or obesity	
		**β coefficient (95% CI)**	***P*-value** [b]	**β coefficient (95% CI)**	***P*-value** [b]	**OR (95% CI)**	***P*-value** [b]
**Birthweight**							
unadjusted model	NBW	Ref.	--	Ref.	--	Ref.	--
	LBW	1.06(-0.24 to 2.36)	0.109	0.48(-0.01 to 0.97)	0.055	1.77 (0.78 to 4.05)	0.175
	HBW	1.52(1.02 to 2.01)	**<0.001**	0.41(0.22 to 0.60)	**<0.001**	1.90 (1.38 to 2.62)	**<0.001**
	P-value [b]	**<0.001**		**<0.001**		**<0.001**	
adjusted model	NBW	Ref.	--	Ref.	--	Ref.	--
	LBW	1.00(-0.86 to 2.86)	0.293	0.39(-0.28 to 1.05)	0.260	0.99(0.23 to 4.28)	0.991
	HBW	1.00(0.40 to 1.59)	**0.001**	0.36 (0.15 to 0.58)	**0.001**	1.22(0.79 to 1.88)	0.377
	P-value [b]	**0.003**		**0.003**		0.674	
**UPF Consumption** [a]							
unadjusted model	Q1	Ref.	--	Ref.	--	Ref.	--
	Q2	0.32(-0.22 to 0.85)	0.243	0.10(-0.10 to 0.29)	0.345	1.21(0.88 to 1.67)	0.233
	Q3	0.48(-0.09 to 1.05)	0.097	0.04(-0.17 to 0.25)	0.679	1.25(0.90 to 1.75)	0.188
	Q4	1.00(0.46 to 1.54)	**0.000**	0.26(0.06 to 0.46)	**0.010**	1.18(0.86 to 1.64)	0.151
	P-value for trend [b]	**0.003**		0.065		0.531	
adjusted model	Q1	Ref.	--	Ref.	--	Ref.	--
	Q2	0.06(-0.64 to 0.75)	0.870	-0.03(-0.28 to 0.22)	0.827	1.11(0.64 to 1.94)	0.709
	Q3	-0.17(-0.90 to 0.56)	0.652	-0.02(-0.29 to 0.24)	0.863	1.18(0.66 to 2.11)	0.571
	Q4	0.05(-0.65 to 0.75)	0.891	0.03(-0.23 to 0.28)	0.845	1.15(0.66 to 1.99)	0.630
	P-value for trend [b]	0.915		0.972		0.948	

Abbreviation: BMI, body mass index; CI, confidence interval; UPF: Ultra-processed foods, Q: quartiles; NBW, normal birthweight; LBW, low birthweight; HBW, high birthweight.

[a] A set of cutoff points for quartiles of UPF consumption was defined at 14.4%, 22.4% and 28.0% of daily food intake.

[b] Generalized linear models were used to investigate associations of birthweight, UPFs consumption with BMI measures in unadjusted models, and further adjusted for child's age and sex (male or female), family income (annual incomes <80,000 RMB, 80,000 to 150.000 RMB, or ≥ 150,000 RMB); maternal education (less than high school, high school and general equivalency, college graduate and above), maternal BMI status (<18.5, 18.5 to 24, 24 to 28, ≥28), physical activity (moderate to vigorous physical activity per day <30 minutes, 30 to 60 minutes, or ≥ 60minutes), child's total daily energy intake (continuous), breastfeeding (Never, <6 month, ≥ 6 months), gestational age (Preterm and early term, full term, late or post-term) and delivery mode (vaginal delivery, caesarean delivery).

NBW or Q1 as reference; *P*-value < 0.05 are in bold type.

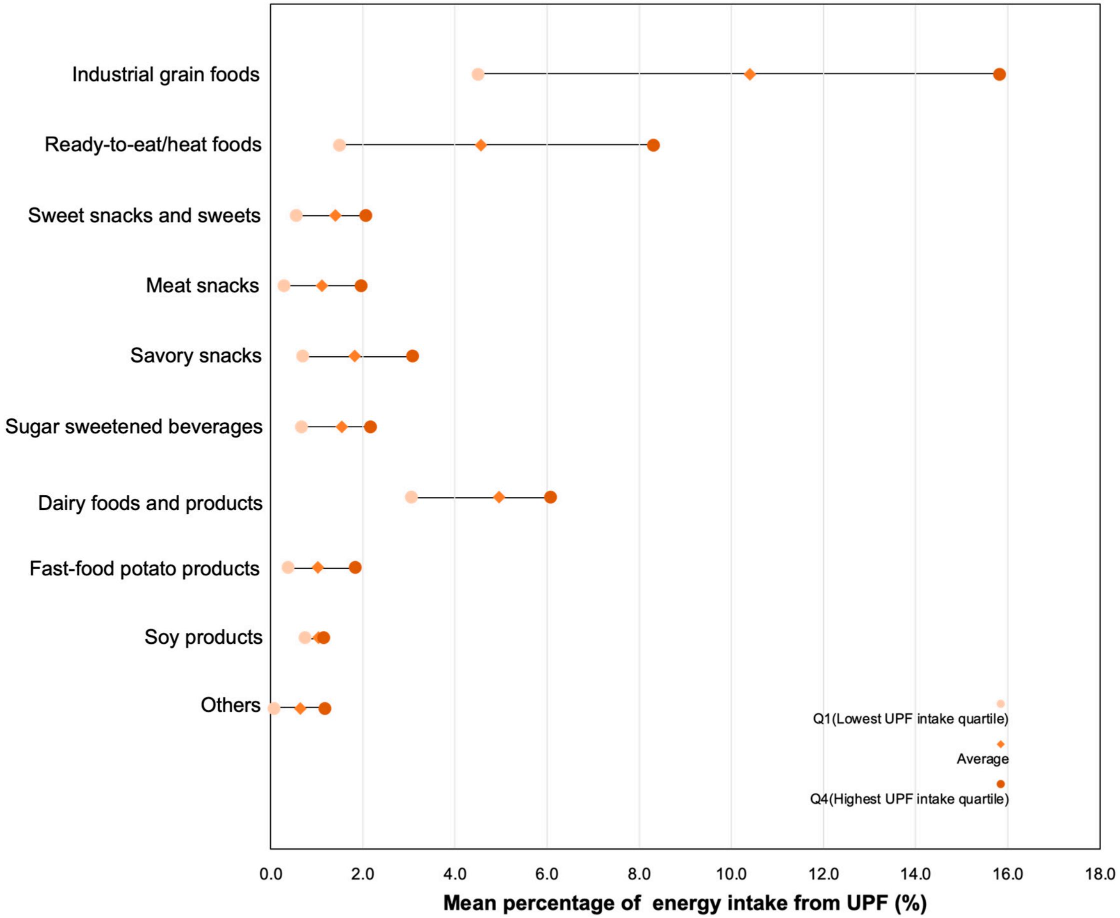

Figure S1. Contributions of food groups to the UPF Consumption Among Children

UPF: Ultra-processed foods, was defined as the proportion of its calorie from consumption of UPF subgroups relative to daily energy intake and was categorized into quartiles (Q1-Q4 represents lowest to highest quartile of percentage of energy intake from UPF consumption).

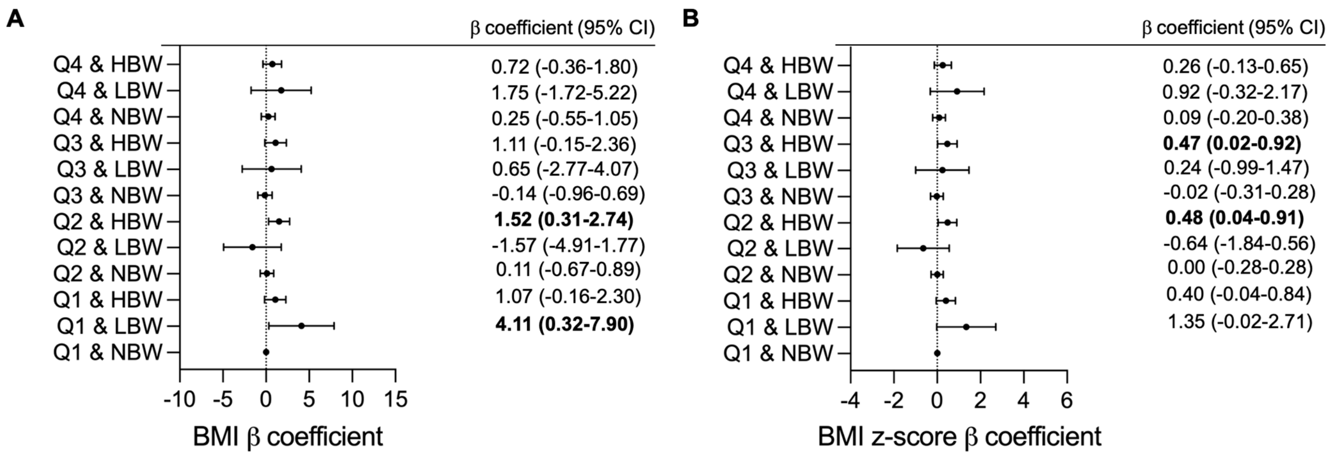

Figure S2. Adjusted β coefficient (95%CI) for childhood BMI measurements according to combined effect of birthweight and UPF consumption

Abbreviation: BMI, body mass index; CI, confidence interval; Q: quartile; NBW, normal birthweight; LBW, low birthweight; HBW, high birthweight.

UPF consumption defined as percentage of UPFs consumption according to the consumption of total daily ultra-processed foods

β coefficient (95%CI) were calculated using a generalized linear model, adjusted for child's age and sex (male or female), family income (annual incomes <80,000 RMB, 80,000 to 150.000 RMB, or ≥ 150,000 RMB); maternal education (less than high school, high school and general equivalency, college graduate and above), maternal BMI status (<18.5, 18.5 to 24, 24 to 28, ≥28), physical activity (moderate to vigorous physical activity per day <30 minutes, 30 to 60 minutes, or ≥ 60minutes), child's total daily energy intake (continuous), breastfeeding (Never, <6 month, ≥ 6 months), gestational age at delivery (Preterm and early term, full term, late or post-term) and delivery mode (vaginal delivery, caesarean delivery). Q1 & NBW as reference; *P*-value < 0.05 are in bold type.
